# Supplementary material for: Improved Light Harvesting of Fiber-Shaped Dye-Sensitized Solar Cells by Using a Bacteriophage Doping Method
Source: Nanomaterials (Basel). 2021 Dec 17;11(12):3421. doi: 10.3390/nano11123421 (PMC8705857; doi:10.3390/nano11123421)
Supplement: Supplementary file 1 [file nanomaterials-11-03421-s001.zip › nanomaterials-1504876-supplementary.pdf]

Supplementary Materials

Improved Light Harvesting of Fiber-Shaped Dye-Sensitized Solar Cells by Using a Bacteriophage Doping Method

Sung-Jun Koo[1,2,†], Jae Ho Kim[1,†], Yong-Ki Kim[3], Myunghun Shin[3], Jin Woo Choi[1], Jin-Woo Oh[2,5,*], Hyung Woo Lee [2,4,*], and Myungkwan Song [1,*]

1 Department of Energy and Electronic Materials, Korea Institute of Materials Science (KIMS), Changwon 51508, South Korea; sungun666@kims.re.kr (S.-J.K.); jho83@kims.re.kr (J.H.K.); jinwoo.choi@kims.re.kr (J.W.C.)
2 Department of Nano Fusion Technology, Pusan National University, Busan 46241, South Korea
3 School of Electronics and Information Engineering, Korea Aerospace University, Goyang 10540, South Korea; ygk3373@kau.kr (Y.-K.M.); mhshin@kau.ac.kr (M.Shin)
4 Department of Nanoenergy Engineering and Research Center of Energy Convergence Technology, Pusan Natuional University, Busan 46241, South Korea
5 Bio-IT Fusion Technology Research Institute, Pusan National University, Busan 46241, South Korea
† These authors contributed equally to this project.
* Correspondence: ojw@pusan.ac.kr (J.-W.O.); LHW2010@pusan.ac.kr (H.W.L); smk1017@kims.re.kr (M.S.)

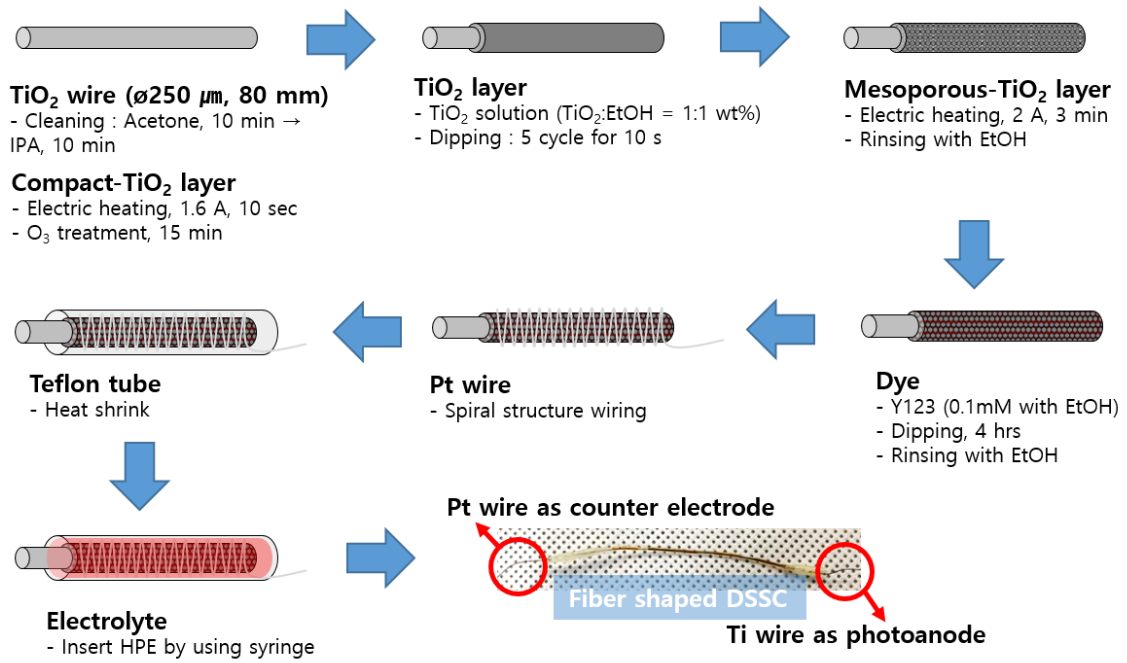

Figure S1. Schematic representation of the fabrication process of FDSSCs.

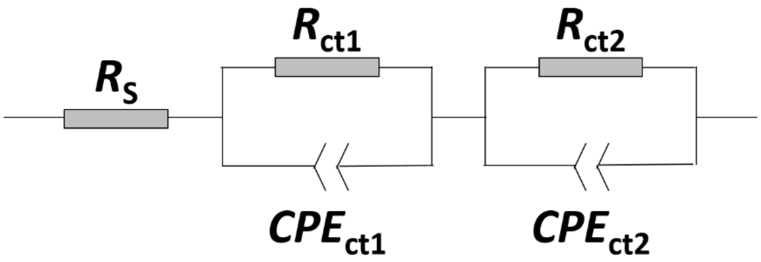

Figure S2. The equivalent circuit used for the EIS analysis.

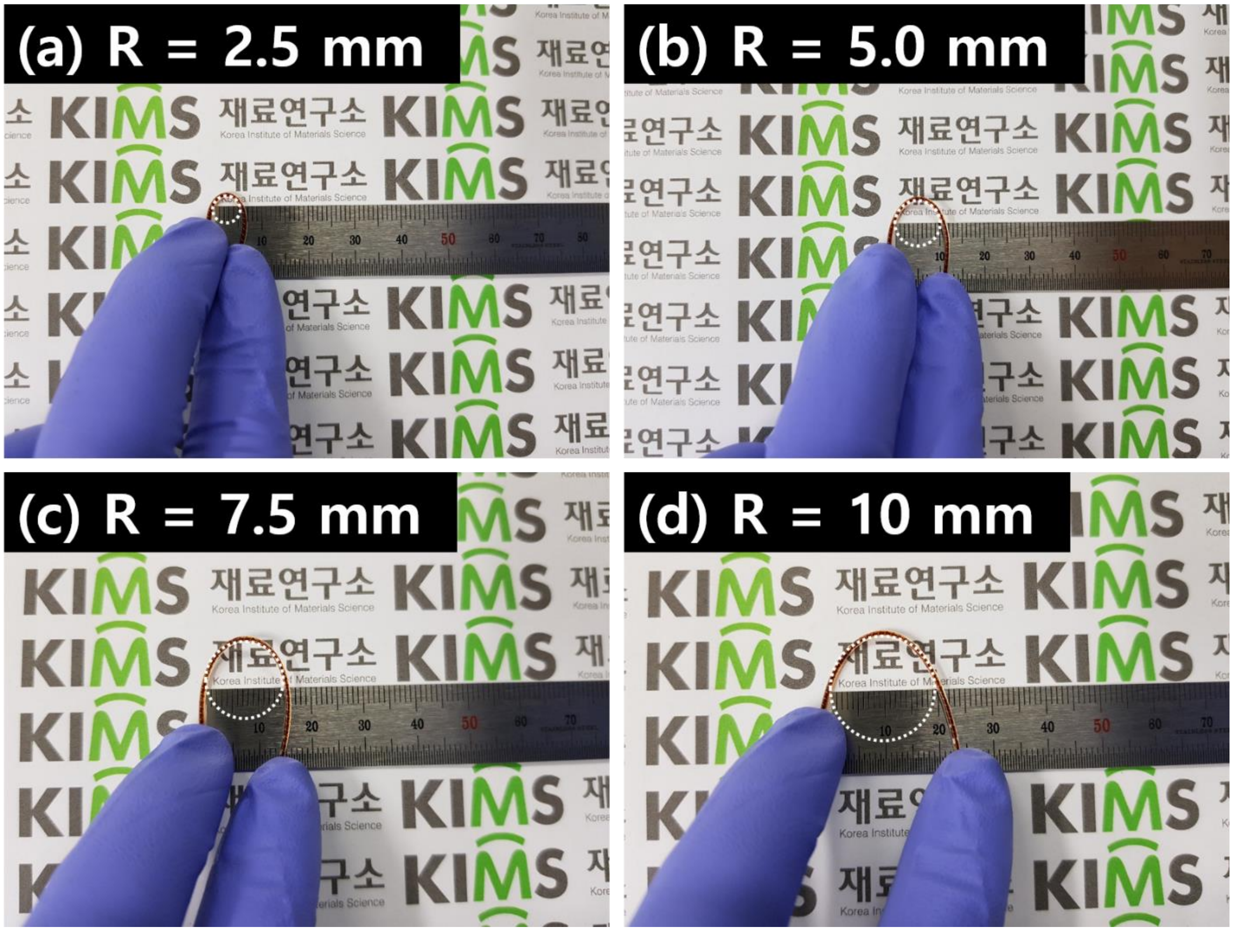

Figure S3. Photographs of Ag@M13 enhanced FDSSCs on bending with width of radius of (**a**) 0.25, (**b**) 0.5, (**c**) 0.75, and, (**d**) 1 cm.

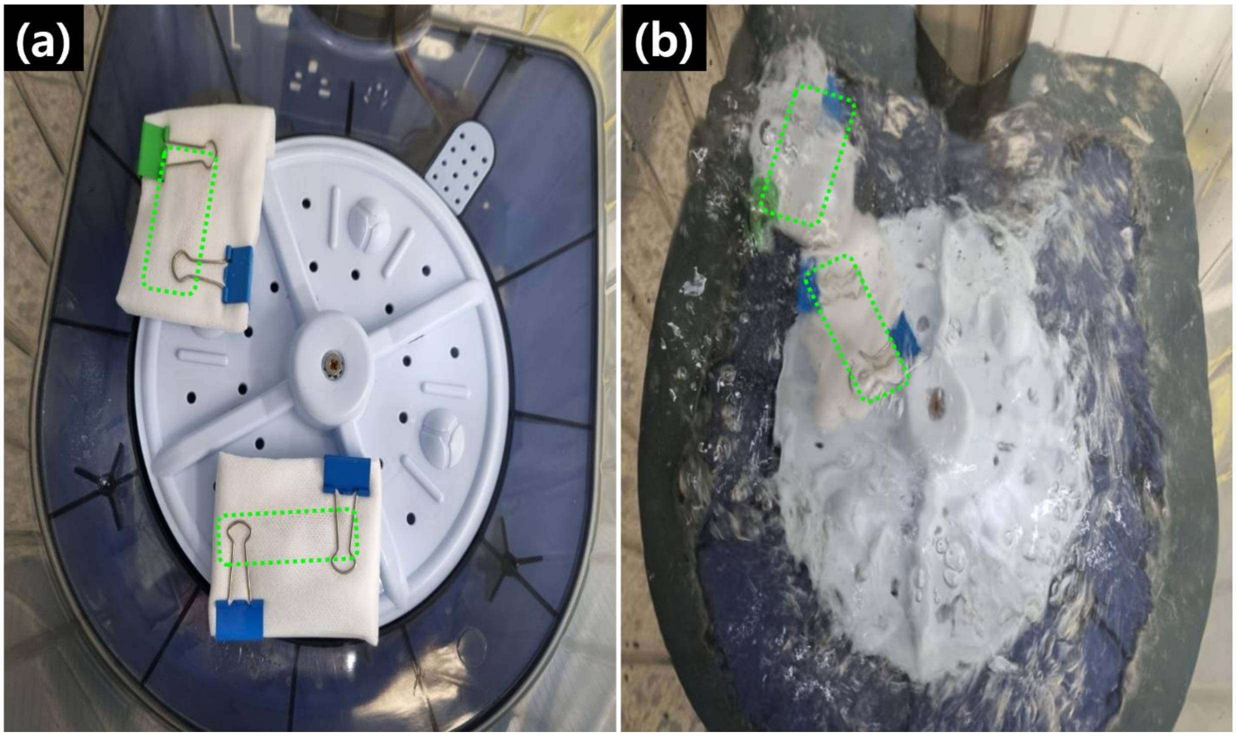

Figure S4. Photographs of Ag@M13 enhanced FDSSCs on being washed in a laundry net: (**a**) the laundry net enclosing the devices was placed in the washing machine, and (**b**) the laundry net and enclosed devices being washed.

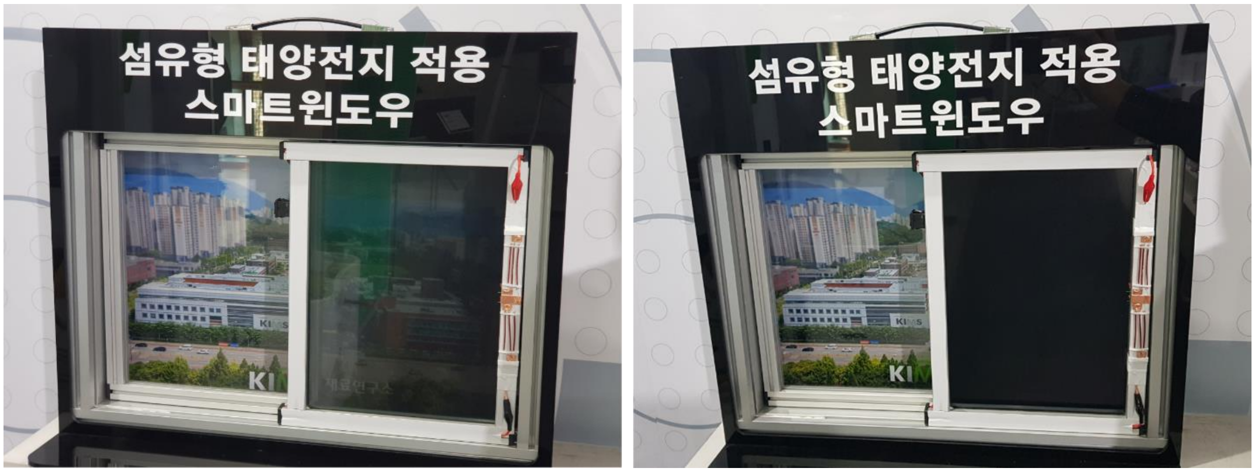

Figure S5. Photographs of Ag@M13 enhanced FDSSCs connected in series and paralled to drive liquid-crystal (LC) device.
